# Donor-Recipient Weight Match in Pediatric Heart Transplantation: Liberalizing Weight Matching with Caution

**DOI:** 10.3390/jcdd9050148

**Published:** 2022-05-07

**Authors:** Ming Chen, Li Xu, Wenjing Yu, Xingyu Qian, Zhenqi Rao, Jingrong Tu, Nianguo Dong, Fei Li

**Affiliations:** 1 Department of Cardiovascular Surgery, Union Hospital, Tongji Medical College, Huazhong University of Science and Technology, Wuhan 430015, China; m202175953@hust.edu.cn (M.C.); unionxuli@hust.edu.cn (L.X.); u201710302@hust.edu.cn (X.Q.); zhenqi_rao@163.com (Z.R.); tjrforwork@hust.edu.cn (J.T.); 2 Key Laboratory of Organ Transplantation, Ministry of Education, NHC Key Laboratory of Organ Transplantation, Key Laboratory of Organ Transplantation, Chinese Academy of Medical Sciences, Wuhan 430015, China; 3 Department of Operating Room, Union Hospital, Tongji Medical College, Huazhong University of Science and Technology, Wuhan 430015, China; wenjing_yu@whxhyy19.wecom.work

**Keywords:** heart transplantation, children, size match, body weight, China

## Abstract

(1) Background: To expand the donor pool, greater donor hearts tended to be used in heart transplantation. However, the data about the feasibility of expanding the donor and recipient weight ratios (DRWRs. All donor and recipient weight ratio (DRWR) in this study or cited from other articles were converted to the DRWR calculated by ((donor weight-recipient weight)/recipient weight) × 100%.) to >30% was still scant in China’s pediatric heart transplantation (HTx). The potential risk increased along with the further expansion of the appropriate range of DRWR to >30% and its upper limit was still in debate. (2) Methods: Seventy-eight pediatric patients (age < 18 years) undergoing HTx between 2015 and 2020 at our center were divided into two groups based on the DRWR (>30% and ≤30%). Variables were summarized and analyzed via univariate analyses and multivariate analyses. A Kaplan-Meier methodology was used to calculate survival and conditional survival. (3) Results: No significant difference was found in one-year, three-year or five-year survival between the two groups. (4) Conclusions: The expansion of DRWR to >30% was acceptable for China’s pediatric HTx. Notably, continuously liberalizing of the upper DRWR boundary to more than 200% could be used as a stop-loss option but should be applied with caution.

## 1. Introduction

In recent decades, greater graft organs were more likely used in heart transplantation (HTx) to expand the door pool. According to the 2019 pediatric HTx International Society for Heart and Lung Transplantation (ISHLT) report, more than 75% of recipients were implanted with a heart, and around 50% were matched by a donor and recipient weight ratio (DRWR) > 30% [1]. Due to the increasingly grim situation of the imbalance between donor and recipient, liberalizing DRWRs was selected as a feasible option to expand the donor pool. According to the 2019 ISHLT report on pediatric HTx, the DRWR > 30% had no impact on the survival of recipients compared with the DRWR ≤ 30%, indicating that a DRWR > 30% had the potential to be applied in the expansion of donor pool [1]. However, data from China was not included in the 2019 ISHLT report, and studies about the field were rarely reported from China.

The appropriate range of DRWRs has been expanded from −30% to 30% in the 2010 ISHLT guideline for adult HTx to −40 to 200% in the 2020 ISHLT consensus for all-age HTx [2,3]. Expanding the range of the DRWR has benefited many patients during the past decade, but whether the DRWR expanded to over 200% was still an appropriate method to solve the limit of donor pool was up for debate, and evidence about the upper limit of expansion were insufficient or had several limitations in pediatric HTx [3,4,5]. Furthermore, Kyle W. Riggs and his colleagues notably indicated that part of those mismatches were caused by the present inequal allocation protocols, in which patients with various weight ranges set forth and the acceptable weight range restrictions were arbitrary in the donor-recipient weight match [5].

Therefore, as the institute with the greatest scale of pediatric heart transplantation in China, we attempted to analyze the prognosis of recipients with a DRWR > 30% by comparing those with a DRWR ≤ 30% to evaluate whether the range of the DRWR > 30% is feasible for expanding the donor pool in China. Since whether the expansion of DRWR more than 30% could still benefit the expansion of the donor pool was still being debated, we explored an appropriate upper limit of the DRWR based on the data from our center combined with reviewing prior relative studies, most likely to provide several suggestions and references for a better utilization of the graft heart under the circumstances.

## 2. Materials and Methods

**Figure 1:**
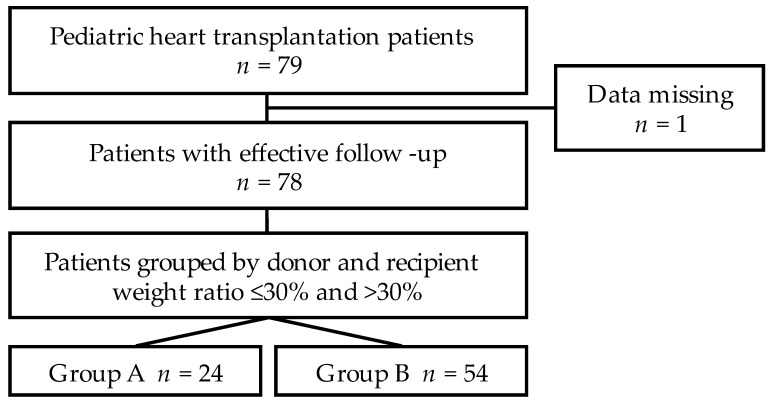
Study cohort design flow chart. All 79 pediatric cardiac transplant patients at Wuhan Union Hospital from January 2015 until December 2020 were recruited. One patient was lost to the follow-up period. A total of 78 recipients were finally enrolled in the study cohort according to the inclusion criteria.

In the present study, we performed a retrospective analysis of pediatric patients (age < 18 years) undergoing heart transplantation. Patients undergoing re-transplantations and recipients with missing data were excluded. The study collected the information on recipients’ and donors’ demographic features and preoperative and postoperative parameters, including survival and postoperative complications. Follow-up information was collected from all survivors either through outpatient visits or telephone investigations with patients or their relatives, and the deadline was 26 May 2021. (Figure 1).

### 2.1. Ethical Statement

All donations after brain death were donated to the Red Cross Society of Hubei Province since 1 January 2015, and were allocated by the China Organ Transplant Response System according to China’s relevant laws and regulations. The Ethics Committee approved the retrospective study protocol of Tongji Medical College of Huazhong University of Science and Technology (IORG No. IORG0003571), the national program for deceased organ donation in China (national protocol for China category I). The clinical and research activities complied with the principles of the Declaration of Istanbul and the Declaration of Helsinki. Mortality data were obtained from the China Heart Transplant Registration Network, where all deaths are registered, as required by law.

### 2.2. Donor-Recipient Ratio

The donor-recipient weight (kg) match was analyzed based on the DRWR. The-78-patient cohort was divided into two groups (Groups A and B) by the DRWR at 30%.

The DRWR was calculated by ((donor weight-recipient weight)/recipient weight) × 100%.

The ranges of the DRWR in each group were as follows:

Group A: DRWR ≤ 30%; Group B: DRWR > 30%; donors weighing greater than the recipient were defined as oversized donors.

The donor-recipient age ratio (DRAR) was calculated by (donor age/recipient age).

### 2.3. Outcome Measures and Definitions

We selected variables of the outcomes of patients as follows: the primary measurements were survival to discharge and survival from operation to final follow up or endpoint; the secondary measurement was the length of hospitalization (days). 

The primary endpoint was an all-cause death event after transplantation. Mortality was obtained from the China Heart Transplant Registration Network, where all deaths were registered, as required by law. 

### 2.4. Statistical Analyses

Demographic data are presented as the median (interquartile range) and mean (standard deviation), where the data were distributed abnormally and normally distributed. Categorical variables are shown as numbers and frequencies (%). Baseline characteristic comparisons, including weight ratio categories, were performed by the chi-square test or Fisher’s precision probability test for continuous variables, and the Mann–Whitney U rank-sum test for categorical variables, as appropriate. The overall survival rates were generated with Kaplan-Meier analysis, and the differences between the two groups based on weight ratio categories were estimated by the log-rank test. Univariate and multivariate Cox proportional hazard regression was used to estimate the hazard ratio (HR) and 95% confidence interval (CI) for factors in OS. In single-factor analysis, a *p* value < 0.2 was considered as a factor for a multivariable analysis. For multivariate analysis, statistical significance was defined by a *p* value of less than 0.05 (two-sided). Analyses was performed by SPSS and R version 4.1 with the packages “survival,” “survminer” and “compareGroups.”

## 3. Results

### 3.1. Demographic Characteristics

From 1 January 2015, to 30 December 2020, 78 children underwent HTx in our center. Table 1 outlines the demographic characteristics of the overall cohort and of each group. 

**Figure 2:**
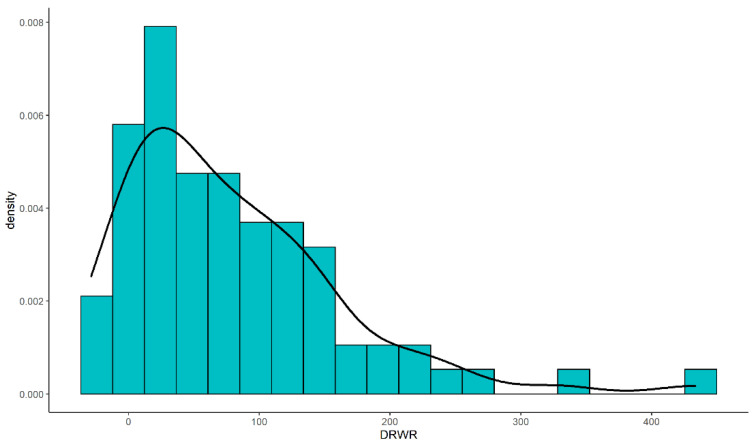
The histograms and density plot of the DRWRs (%). The DRWRs among the recipients concentrated in the range of 0 to 100%.

The age of the recipient, weight of the recipient, DRWR, DRAR, diagnosis of the recipients and preoperative angiotensin receptor blocker (ARB) were significantly different between the two groups. Other variables showed no significant difference. (Table 1). The distribution of the DRWRs of the recipients were demonstrated in Figure 2.

Of all patients undergoing HTx, 69.2% patients had a DRWR over 30%, and the median of DRWR was at 63.7% [16.8; 122]. In addition, a greater level of weight mismatch (the median of DRWR was 5.80 [−9.10; 13.6] and 98.5 [60.7; 140] in Group B) could be observed in younger patients with a mean age of 8.35 (±4.46) years old (*p* < 0.001) and in lighter patients with a median bodyweight of 24.2 [14.0; 33.0] kg (*p* < 0.001) in Group B. The same could also be seen in those patients with a greater donor-to-recipient age ratio (DRAR) (*p* < 0.001). 

The diagnosis was significantly different between the two groups (*p* = 0.001); for example, dilated cardiomyopathy (DCM) accounted for the major proportion (57/78, 73.1%) of diagnoses, with 11/24 (45.8%) in Group A, and 46/54 (85.2%) were in Group B.

### 3.2. Post-Transplantation Outcomes

Regarding postoperative outcomes, respiratory complications were the major complications (56.4%), and the morbidity of respiratory complications in Group A and Group B were 50.0% and 59.3%, respectively (*p* = 0.607). There was no significant difference between the two groups in neurological complications (*p* = 1), renal complications (*p* = 1), or liver damage (*p* = 0.05).

The length of postoperative hospitalization (LOPH) was significantly different between the two groups: the LOPH (53.5 [39.2; 72.8]) in Group B was vastly more prolonged than that in Group A (36.0 [29.0; 43.5]).

**Figure 3:**
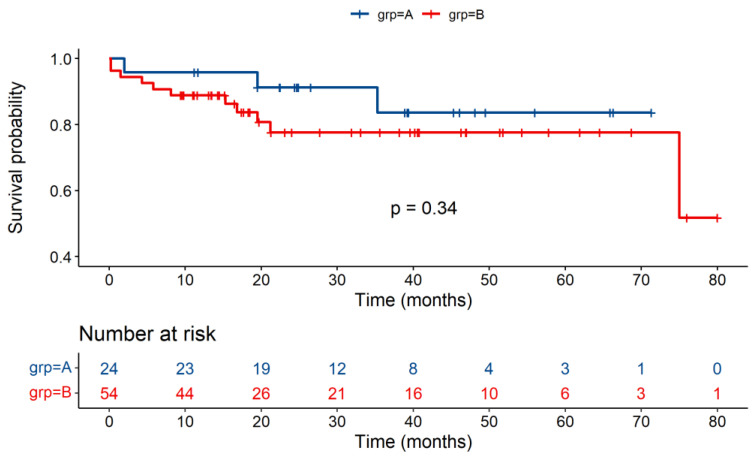
Overall Survival of Recipients in Group A and Group B. Kaplan–Meier estimates of survival after heart transplantation with log-rank test, comparing the outcomes between Group A (DRWR ≤ 30%) and Group B (DRWR > 30%), log-rank *p* = 0.34.

During the period from operation to discharge, the overall mortality was 17.9% (14/78), and mortality was 12.5% (3/24) in Group A and was 20.4% (11/54) in Group B, showing no significant difference between the two groups (*p* = 0.531). The in-hospital mortality was almost the same, with 4.17% in Group A and 7.41% in Group B (*p* = 1.000). No significant difference in survival between the two groups was observed at different times or in the K-M curve. (Table 2 and Table 3, and Figure 3).

### 3.3. Detailed Information about Recipients with DRWR > 200%

Seven patients with a DRWR greater than 200% were included (6.4%), and the greatest DRWR in the cohort was 433.3%. The average age of the recipients with a DRWR > 200% (10.2 years) was lower than that in normal group (5.00 years). The mortality of those recipients with a DRWR > 200% was higher (42.9%) than those ≤200% (5.6%), while all the data of the recipients with a DRWR > 200% was so small that was not able to be statistically compared. All of the death events of those recipients happened in a short period after transplantation. The DRWR and age of the three dead cases were 212.5 (eight years), 257.1 (four years) and 433.3 (one year), respectively. The latter two cases died in the ICU within seven days after transplantation due to acute rejection and malignant arrhythmia, respectively, while severe liver injury and reparatory complications accompanied by systematic infection were the causes of death in the first case (Table 4 and Table 5).

Hematologic data and the use of anti-rejection drugs of the seven patients with DRWR > 200% after HTx were demonstrated in Supplementary Materials.

### 3.4. Univariate and Multivariate Analyses

In univariate analysis, variables included recipient blood type, DRWR, DRAR, blood-type match, preoperative ACEI, preoperative BB, and respiratory complications. Univariate and multivariate analyses based on the variables mentioned above showed that there was no risk factor for the overall death. 

## 4. Discussion

There was an increased application of oversized donors in pediatric HTx, and the liberalization of the range of the donor-recipient weight match has long been considered as an approach to expand the donor pool [6]. The present study analyzed the feasibility of the range expansion to a DRWR > 30% in China’s pediatric HTx and made a further exploration about the upper bound of the expansion of the DRWR based on the data from our center and prior studies to provide suggestions and supplemental methods to balance the expanding donor pool and the utilization of graft hearts at the stage of pre-transplantation size-matching.

### 4.1. DRWR > 30% Is Feasible in China

The major finding in the present study was that the DRWR > 30% was not associated with high mortality in terms of one-year, three-year, and five-year survival (*p* = 0.33, 0.34, 0.34, respectively). 

Poorer survival and the higher morbidity of postoperative complications of undersized donors were proven in prior studies, and the organs of oversized donors showed better tolerance for complex environments [2,7,8]. In the size match of allograft organs, oversized donors were more easily matched because of the greater donor pool compared with undersized donors. Therefore, an oversized donor was largely used in the size match of pediatric HTx, and the donor weight showed a similar distribution in this study.

The 2010 ISHLT guideline of HTx recommended that a DRWR −30–30% was uniformly safe for all-ages HTx [2]. However, this recommendation referred to two articles issued the 1990s. With the development of surgical techniques and apparatus in recent decades, many successful transplantations cases performed with a DRWR > 30% have been reported. According to the 2019 ISHLT report about pediatric HTx, mid-term (five-year) and long-term (10-year) survival showed no difference among different age groups and weight groups [1]. For short-term (one-year) survival, however, only recipients aged from one to five years showed better survival when matched by a DRWR > 30% compared with a DRWR < −30%. In line with the report, one-year, three-year and five-year survival showed no significant difference between recipients with a DRWR ranging from −29.3% to 30% and those larger than 30%. Besides, the latest consensus suggested that a DRWR range from −40% to 200% was a safe zone for pediatric HTx [3]. Therefore, it is believed that a DRWR > 30% would be safely applied to expand the donor pool in Chinese pediatric HTx.

### 4.2. Expansion of the DRWR to Over 200% Is Potentially Risky

In the past decade, the expansion of DRWRs indeed benefited many recipients. However, several risks increased with the further expansion of DRWR to >200%. 

In the present study, the mortality (42.9%) increased when the range of the DRWR expanded to >200%, and all the death events of those recipients with DRWR > 200% happened in a short period after transplantation, though the sample size of those recipients was so small that was not able to be statistically compared. To date, several studies proved that the feasibility of the DRWR ranged from 30% to 200%, while only a few reports have focused on weight mismatch for DRWR > 200%. Several previous studies showed that the DRWR > 200% had no obvious impact on the survival of recipients. A large-scale study, which divided recipients by a DRWR = 150%, reported that the one-year, three-year, and five-year survival rates were not significantly different in pediatric recipients with congenital heart disease [9]. A large multicenter study by Kyle W. Riggs et al. showed that recipients with variable DRWRs (median DRWR 100%, range 59% to 241%) had similar survival rates after transplantation, and critically ill recipients could benefit from a higher DRWR (≥100%), while those with a DRWR > 200% were not enrolled in this study [5]. A single center study of Mazyar K. and his colleagues indicated that no significant difference in the survival was found between the survival of recipients with a DRWR > 200% (*n* = 9) and those with a DRWR ≤ 200% (*n* = 126). It concluded that a DRWR > 200% could be safely used in expanding the donor pool, though the number of recipients with DRWR > 200% was too small to make a strong conclusion [4]. On the contrary, in the present study, recipients with a DRWR > 200% showed a high mortality of 42.9% (3/7), while the number of those recipients with a DRWR > 200% was also small. In addition, the 2010 ISHLT guidelines suggested that a DRWR > 100% was an independent risk factor for primary graft failure [2]. The latest ISHLT consensus of pediatric HTx in 2020 suggested that a DRWR from −40% to 200% had no adverse effect on outcomes [3]. However, it is noteworthy that the upper limit of the range in the consensus was based on the single center research of Mazyar K. et al., and its limitation was mentioned above [4]. Therefore, the upper boundary of the DRWR in pediatric HTx is still controversial. 

The data in this study showed that there was a relatively lower average age and higher mortality in recipients with a DRWR > 200% than those in ≤200%, though they were unable to be statistically compared. This difference also suggested that younger recipients were prone to be matched with an allograft organ from a donor with an extremely greater heart (or a higher DRWR), which was generally accompanied by delayed sternal closure (DSC). Although it was reported as a beneficial adjunct in the open chest surgery and in the initial postoperative period, DSC would trigger poor physical condition and high morbidity of postoperative complications [9,10]. However, given the limitation of the small sample size in the DRWR > 200%, it is difficult to draw any strong conclusion regarding the outcomes of this group. 

Therefore, given the risks and the uncertainty of the effects on outcomes, donors with DRWR > 200% should be utilized in the practical expansion of the donor pool with more caution in pediatric HTx. 

### 4.3. The Outlooks of the Methods to Improve the Utilization of Graft Organ When DRWR > 200% in the Pre-Transplantation Donor-Recipient Size Matching

Of note, a larger donor pool before HTx and a better outcome after pediatric HTx are the primary objectives to expand the boundary of the DRWR. Therefore, ways to balance the two primary goals, even though there were several conflicts between them, were necessary to be discussed.

The restriction of the range of donor-recipient size matches might be able to hedge the risks when the DRWR > 200%, but it would also lead to a long period on the waiting-list, which would inevitably be associated with poor physical condition and cardiopulmonary function. Thus, though DRWR > 200% is a risky zone for pediatric HTx, it is still a stop-loss option for patients. 

On the basis of this dilemma, it is necessary to control risks in further studies under this circumstance, such as setting rigorous treatment and postsurgical surveillance strategies for a recipient with a DRWR > 200%. However, a rigorous inclusion criterion seems to be an applicable solution for improving the utilization of allografts, but it contradicts the original intention—expanding the donor pool. In this respect, Kyle W. Riggs and his colleagues concluded that part of those mismatches was caused by the present inequal allocation protocols, in which patients with various weight ranges set forth and the acceptable weight range restrictions were arbitrary. Thus, they proposed some possible solutions for better organ use, including normalizing the criteria programs use and using supplemental methods for size-match analysis [5]. 

Different matching methods of the donor-to-recipient match could be a supplemental option. Prior studies have explored many other indicators, such as height, body mass index (BMI) mismatch, ejection fraction (EF), and predicted heart mass (PHM) mismatch, in order to find a more suitable metric for matching in adult HTx [3,11,12,13,14]. However, pediatric HTx has lagged in this field. Meanwhile, previous studies demonstrated a direct and accurate donor and recipient match method, known as “virtual transplantation”, for expanding the donor upper limit of the size match, which was a direct visual confirmation of the donor-to-recipient organ size match using cross-sectional imaging in pediatric HTx [15,16,17]. However, it was limited by information accessibility. Therefore, different matching methods could be used to improve the utilization of the graft organ and the outcomes after HTx for recipients with a DRWR > 200%. 

Mechanical circulation support (MCS), including ventricular assist devices (VAD) and total artificial heart (TAH), is also an alternative approach before a fitting graft organ transplantation, instead of implanting an extreme oversized heart. After decades of development, MCS was increasingly being used as a bridge to transplant (BTT) in pediatric HTx, and current research results showed a promising future of MCS in pediatric HTx. Several recently published studies indicated that the use of VAD as BTT showed a better prognosis after pediatric HTx compared with the pre-operative non-VADs [18,19]. A newly created TAH, BiVACOR, is small enough that it can be implanted in a child [20]. Future exploration in MCS used as an alternative way of expanding the donor pool to DRWR > 200% was required.

## 5. Conclusions

A DRWR range of >30% was feasible for the size match for pediatric HTx. The liberalization of the weight match was a potential approach to increase the donor pool, but the risks associated with a DRWR ≥ 200% were proven to be uncertain for pediatric HTx. Consequently, strategies to improve the donor pool by liberalizing the upper DRWR boundary to more than 200% could be used as a stop-loss option, but should be applied with more caution.

## 6. Limitations

Initially, the limitations of this study were principally those biases that any retrospective study has, and the accuracy of the study was in line with the accurate data collection around the event. Moreover, even though the data were collected from the largest pediatric HTx center in China, this was still a single-center study and the sample size was limited, as only 78 patients were available during our follow-up. In addition, the follow-up time was relatively short, since only short-term (one-year) and middle-term (five-year) survival rates were included.

## Figures and Tables

**Table 1:** Baseline characteristics.

Variables	All Patients	Group A DRWR ≤ 30%	Group B DRWR > 30%	*p* Value
Numbers	*n* = 78	*n* = 24	*n* = 54	
Recipient sex (%)				0.925
Male	40 (51.3%)	13 (54.2%)	27 (50.0%)	
Female	38 (48.7%)	11 (45.8%)	27 (50.0%)	
Recipient age, year	9.74 (4.70)	12.9 (3.67)	8.35 (4.46)	<0.001
Recipient weight, kg	30.0 [17.1; 42.5]	48.5 [39.5; 53.0]	24.2 [14.0; 33.0]	<0.001
Recipient blood type (%)				0.356
A	21 (26.9%)	9 (37.5%)	12 (22.2%)	
B	29 (37.2%)	6 (25.0%)	23 (42.6%)	
AB	25 (32.1%)	8 (33.3%)	17 (31.5%)	
O	3 (3.85%)	1 (4.17%)	2 (3.70%)	
Donor age, years (%)	18.5 [11.0; 30.2]	18.5 [12.8; 24.5]	18.5 [8.50; 31.8]	0.828
Donor sex (%)				0.651
Male	50 (64.1%)	14 (58.3%)	36 (66.7%)	
Female	28 (35.9%)	10 (41.7%)	18 (33.3%)	
Donor weight, kg (%)	50.0 [35.2; 60.0]	50.0 [40.0; 55.0]	50.0 [31.2; 60.0]	0.493
DRWR (%)	63.7 [16.8; 122]	5.80 [−9.10; 13.6]	98.5 [60.7; 140]	<0.001
DRAR	1.81 [1.33; 2.73]	1.39 [1.00; 1.68]	2.20 [1.44; 3.50]	<0.001
Sex identical match (%)	34 (43.6%)	9 (37.5%)	25 (46.3%)	0.634
Blood-type identical match (%)	40 (51.3%)	12 (50.0%)	28 (51.9%)	1
Diagnosis (%)				0.001
DCM	57 (73.1%)	11 (45.8%)	46 (85.2%)	
CAD	2 (2.56%)	2 (8.33%)	0 (0.00%)	
VHD	4 (5.13%)	3 (12.5%)	1 (1.85%)	
CHD	14 (17.9%)	7 (29.2%)	7 (13.0%)	
Others	1 (1.28%)	1 (4.17%)	0 (0.00%)	
Cardiac surgery history (%)	18 (23.1%)	9 (37.5%)	9 (16.7%)	
Cold ischemia time, minutes	354 [320; 375]	348 [322; 374]	364 [320; 375]	0.439
Preoperative IABP (%)	1 (1.28%)	1 (4.17%)	0 (0.00%)	0.308
Preoperative ECMO (%)	5 (6.41%)	1 (4.17%)	4 (7.41%)	1
Preoperative dopamine (%)	46 (59.0%)	15 (62.5%)	31 (57.4%)	0.863
Preoperative ACEI (%)	32 (41.0%)	9 (37.5%)	23 (42.6%)	0.863
Preoperative ARB (%)	7 (8.97%)	5 (20.8%)	2 (3.70%)	0.026
Preoperative BB (%)	28 (35.9%)	12 (50.0%)	16 (29.6%)	0.14

Values are presented as percentages in parentheses () and as lower and upper quartiles in brackets []. DRWR, donor-recipient weight ratio calculated by ((donor weight-recipient weight)/recipient weight) × 100%; DRAR, donor/recipient age ratio calculated by donor age/recipient age; DCM, dilated cardiomyopathy; CAD, coronary artery disease; VHD, valve heart disease; CHD, congenital heart disease; IABP, the intra-aortic balloon pump; ECMO, extracorporeal membrane oxygenation; ACEI: angiotensin converting enzyme inhibitors; ARB: angiotensin receptor blocker; BB: β-receptor blocker.

**Table 2:** Outcomes after heart transplantation.

Variables	All Patients	Group A DRWR ≤ 30%	Group B DRWR > 30%	*p* Value
Number	*n* = 78	*n* = 24	*n* = 54	
**Early Postoperative Data**				
Pneumonia (%)	44 (56.4%)	12 (50.0%)	32 (59.3%)	0.607
Neurological complications (%)	6 (7.69%)	2 (8.33%)	4 (7.41%)	1
Acute renal injury (%)	1 (1.28%)	0 (0.00%)	1 (1.85%)	1
Liver injury (%)	9 (11.5%)	0 (0.00%)	9 (16.7%)	0.05
Sepsis (%)	0 (0%)	0 (0%)	0 (0%)	-
LOPH, days	44.5 [33.2; 66.2]	36.0 [29.0; 43.5]	53.5 [39.2; 72.8]	0.002
In-hospital death (%)	5 (6.41%)	1 (4.17%)	4 (7.41%)	1
**Long-Term Postoperative Data**				
Death (%)	14 (17.9%)	3 (12.5%)	11 (20.4%)	0.531
Length of survival, months	23.6 [14.4; 45.9]	30.9 [22.4; 46.6]	19.6 [13.2; 40.8]	0.075

Values are presented as percentages in parentheses () and as lower and upper quartiles in brackets []. Pneumonia, diagnostic criteria: cough with sputum plus the detection of bacteria by sputum culture or high-throughput sequencing; Neurological complications include epilepsy, intracranial infection, cerebrovascular hemorrhage, cerebral ischemia and hemiplegia; Acute renal injury, diagnostic criteria: the creatinine concentration (CC) ≥ 0.3 mg/dL or/and urine output < 0.5 mL/kg/h for 6–12 h; Acute liver injury, diagnostic criteria: ALT > 3 times of the baseline. Sepsis and septic shock, diagnostic criteria: Increase in the Sequential Organ Failure Assessment (SOFA) score ≥ 2 from baseline or quick SOFA ≥ 2 and suspected infection; LOPH, the length of postoperative hospitalization (days).

**Table 3:** Survival after heart transplantation.

Survival	Group A	Group B	*p* Value
1-year survival%	95.8	88.9	0.33
3-year survival%	83.7	77.6	0.34
5-year survival%	83.7	77.6	0.34
Overall survival%	83.7	51.8	0.34

**Table 4:** Detailed baseline characteristics of patients with a DRWR > 200%.

Patients	Patient A	Patient B	Patient C	Patient D	Patient E	Patient F	Patient G
Diagnosis	DCM	DCM	DCM	DCM	DCM	DCM	DCM
Recipient sex	female	female	female	male	male	male	female
Recipient age, year(s)	1	6	5	10	1	8	4
Recipient weight, kg	8.3	14	14.5	28	7.5	16	14
Recipient blood type	AB	AB	AB	B	A	A	B
Donor age, years	8	10	33	35	13	18	32
Donor sex	male	male	male	male	female	female	female
Donor weight, kg	25	60	50	90	40	50	50
DRWR (%)	201.2	328.6	244.8	221.4	433.3	212.5	257.1
DRAR	8	1.67	6.6	3.5	13	2.25	8
Cold ischemia time, minutes	370	345	365	394	394	348	498
Blood-type identical match	√			√			
Cardiac surgery history							
Preoperative IABP							
Preoperative ECMO		√					√
Preoperative dopamine		√	√	√	√		√
Preoperative ACEI	√	√	√	√	√		
Preoperative ARB			√	√			
Preoperative BB		√	√	√		√	
RBC, 10^12^	4.94	4.16	2.15	4.27	4.32	5.92	4.02
PLT, 10^9^	404	327	97	270	314	281	257
WBC, 10^9^	7.77	5.68	6.42	9.29	3.89	11.52	12.22
Neut (%)	83.8	58.8	77.88	50.81	20.82	64.93	57.69
Lym (%)	50.8	36.62	10.9	39.4	64.27	23.78	35.68
ALT, U/L	12	15	909	10	26	32	29
AST, U/L	36	24	1557	23	64	49	37
Tbil, mmol/L	10.3	7.7	23.1	8.9	9.3	20	9.8
SCr, μmol/L	26.3	52.1	33.8	50.1	33.1	48.7	41.3
BUN, μmol/L	3.5	9.7	6.63	5.8	5.27	5.92	7.3

Values are presented as percentages in parentheses () and as lower and upper quartiles in brackets []. DRWR, donor-recipient weight ratio calculated by ((donor weight-recipient weight)/recipient weight) × 100%; DRAR, donor-recipient age ratio calculated by donor age/recipient age; DCM, dilated cardiomyopathy; CAD, coronary artery disease; VHD, valve heart disease; CHD, congenital heart disease; IABP, the intra-aortic balloon pump; ECMO, extracorporeal membrane oxygenation; ACEI, angiotensin converting enzyme inhibitors; ARB, angiotensin receptor blocker; BB, β-receptor blocker.

**Table 5:** Detailed outcomes of recipients with a DRWR > 200% after heart transplantation.

Early Postoperative Data	Patient A	Patient B	Patient C	Patient D	Patient E	Patient F	Patient G
Pneumonia (%)	√	√	√			√	
Neurological complications (%)							
Acute renal injury (%)							
Liver injury (%)		√	√			√	
Sepsis (%)							
In-hospital death					√	√	√
LOPH, days	54	51	41	77	6	46	7
Death					√	√	√
Cause of death					Malignant arrhythmia	Systemic infection	Acute rejection

Pneumonia, diagnostic criteria: cough with sputum plus the detection of bacteria by sputum culture or high-throughput sequencing; Neurological complications include epilepsy, intracranial infection, cerebrovascular hemorrhage, cerebral ischemia and hemiplegia; Acute renal injury, diagnostic criteria: the creatinine concentration (CC) ≥ 0.3 mg/dL or/and urine output < 0.5 mL/kg/h for 6–12 h; Acute liver injury, diagnostic criteria: ALT > 3 times of the baseline. Sepsis and septic shock, diagnostic criteria: Increase in the Sequential Organ Failure Assessment (SOFA) score ≥ 2 from baseline or quick SOFA ≥ 2 and suspected infection; LOPH, the length of postoperative hospitalization (days).

## Data Availability

All date were included in this manuscript.

## Supplementary Materials

The following are available online at https://www.mdpi.com/article/10.3390/jcdd9050148/s1, Figure S1: Hematologic data and the use of anti-rejection drugs of the seven patients with DRWR > 200% after HTx.
