# Supplementary material for: Donor-Recipient Weight Match in Pediatric Heart Transplantation: Liberalizing Weight Matching with Caution
Source: J Cardiovasc Dev Dis. 2022 May 7;9(5):148. doi: 10.3390/jcdd9050148 (PMC9145031; doi:10.3390/jcdd9050148)
Supplement: Supplementary file 1 [file jcdd-09-00148-s001.zip › jcdd-1573069-supplementary.pdf]

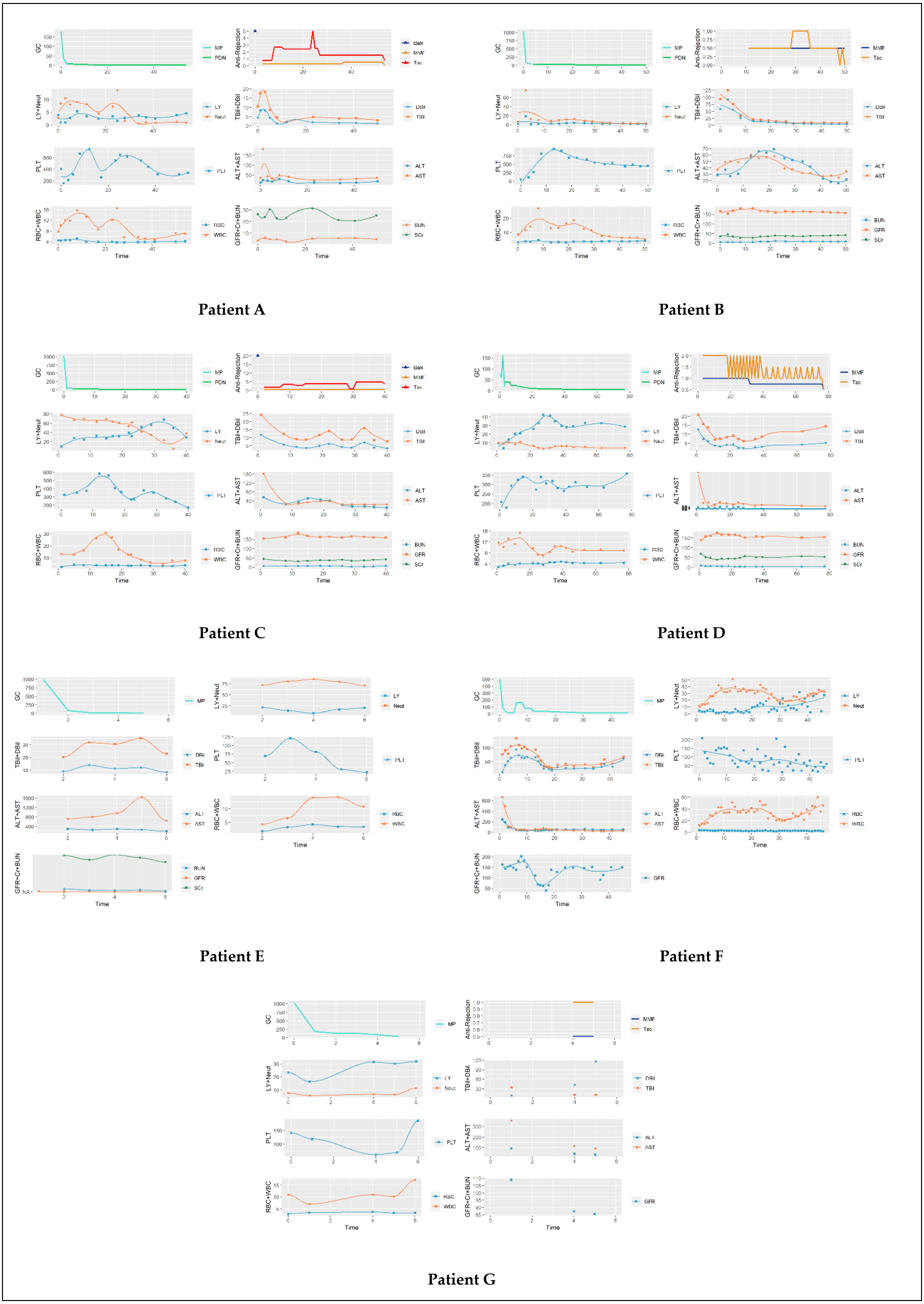
Patient A
Patient B
Patient C
Patient D
Patient E
Patient F
Patient G

Figure S1. Hematologic data and the use of anti-rejection drugs of the seven patients with DRWR>200% after HTx.

RBC, red blood cell; PLT, platelet; WBC, white blood cell; Neut, neutrophils; Lym, lymphocyte; ALT, alanine aminotransferase; AST, aspartate aminotransferase; Tbil, total bilirubin; SCr, serum creatinine; BUN, blood urea nitrogen.
